# Dynamic Monitoring of Recurrent Ovarian Cancer Using Serial ctDNA: A Real-World Case Series

**DOI:** 10.3390/curroncol32100585

**Published:** 2025-10-21

**Authors:** Eric Rios-Doria, Jonathan B. Reichel, Marc R. Radke, Enna Manhardt, Mayumi Rubin-Saika, Christina Lockwood, Elizabeth M. Swisher, Kalyan Banda

**Affiliations:** 1 Division of Gynecologic Oncology, Department of Obstetrics and Gynecology, University of Washington, Seattle, WA 98195, USA; riosdore@uw.edu (E.R.-D.); mrradke@uw.edu (M.R.R.); ennahun@uw.edu (E.M.); mayumirs@uw.edu (M.R.-S.); swishere@uw.edu (E.M.S.); 2 Department of Laboratory Medicine and Pathology, University of Washington, Seattle, WA 98195, USA; jonreich@uw.edu (J.B.R.); tinalock@uw.edu (C.L.); 3 Brotman Baty Institute for Precision Medicine, University of Washington, Seattle, WA 98195, USA; 4 Clinical Research Division, Fred Hutchinson Cancer Center, Seattle, WA 98109, USA; 5 Division of Hematology and Oncology, Department of Medicine, University of Washington, Seattle, WA 98109, USA

**Keywords:** ovarian cancer, circulating tumor DNA, ctDNA, liquid biopsy, minimal residual disease, recurrence, next-generation sequencing, personalized oncology, biomarkers

## Abstract

Recurrent ovarian cancer (OC) is challenging to detect early using current methods like CA-125 and imaging. Circulating tumor DNA (ctDNA) may improve disease monitoring. Here, we assess the real-world clinical utility of serial ctDNA analyses in patients with recurrent OC. We analyzed serial plasma samples (*N* = 23) from six patients with recurrent OC using a tumor-informed next-generation sequencing assay targeting 68 cancer-related genes developed at the University of Washington. ctDNA variant allele frequencies (VAFs) were correlated with CA-125 levels, radiographic findings, and clinical outcomes. ctDNA levels generally reflected clinical status, accurately mirroring disease progression and therapeutic response. In one patient, rising ctDNA preceded clinical recurrence by four months, despite normal CA-125 and imaging, highlighting its potential advantage. Conversely, some patients exhibited clinical progression with undetectable ctDNA, indicating limitations in assay sensitivity, biological factors, or metastatic sites (e.g., brain metastases). ctDNA and CA-125 showed complementary value in most cases, suggesting potential combined use in clinical monitoring. Our findings demonstrate that ctDNA is a promising biomarker to complement existing monitoring approaches for recurrent OC. In some cases, capable of predicting relapse and treatment response ahead of current clinical indicators. However, identified discordances underscore technical and biological challenges that warrant further investigation. Larger prospective studies are necessary to refine ctDNA’s clinical utility and integration into personalized OC care.

## Simple Summary

Ovarian cancer often comes back after the first treatments, but it is hard to detect and monitor in time. Tests like CT scans and blood tests (CA-125) are not always accurate in showing if or when the cancer returns or grows. In this study, we tested whether measuring pieces of cancer DNA found in the blood (circulating tumor DNA or ctDNA) could help detect ovarian cancer earlier and better. We collected serial blood samples from patients over time and found that changes in ctDNA usually matched closely with whether the patient’s cancer was growing or responding to treatment. In one case ctDNA even gave warnings of the cancer returning many months before it could be seen on CT scans or blood tests. However, in a few cases, ctDNA did not show cancer growth. These results suggest that using ctDNA could help make personalized decisions about treatment in ovarian cancer.

## 1. Introduction

Ovarian cancer remains a major therapeutic challenge due to its propensity for late-stage presentation, high rates of recurrence, and eventual development of chemotherapy-resistant disease. In recent years, circulating tumor DNA (ctDNA) has garnered significant attention as a potential biomarker, offering a non-invasive method to detect minimal residual disease, monitor treatment response, and identify recurrence earlier than conventional radiographic or clinical assessments in several solid tumor types, including melanoma, colorectal, and breast cancers [1,2,3]. Unlike traditional tissue biopsy, ctDNA assesses all genetic alterations within a given tumor, including clonal and subclonal mutations [4]. A traditional tissue biopsy is limited in identifying this tumoral heterogeneity. Within other cancer types, the use and integration of ctDNA into clinical practices and clinical trials is expanding [5,6]. Moreover, when compared to patients with lower levels of ctDNA, those with higher levels may have a less favorable treatment response or survival benefit [7,8,9].

In ovarian cancer, ctDNA has shown promise for early detection of recurrence—sometimes preceding radiographic findings by several months—and for monitoring treatment response. However, its utility is not yet established. Key controversies remain: not all ovarian cancers shed detectable ctDNA, particularly those with low-volume or microscopic disease, raising concerns about false-negative results. The sensitivity of ctDNA varies by tumor burden, site of disease, and assay methodology. Furthermore, whether earlier detection via ctDNA meaningfully improves outcomes, given current treatment options, remains uncertain. Technical challenges also exist. Highly sensitive sequencing or digital PCR is required to detect rare ctDNA fragments amid abundant normal cell-free DNA. False positives may occur due to sequencing artifacts or clonal hematopoiesis. Conversely, false negatives can arise from low-shedding tumors or suboptimal assay design. Identifying tumor-specific markers and standardizing testing approaches will be critical to advancing clinical implementation.

Our study presents a real-world case series of six ovarian cancer patients undergoing serial ctDNA testing across multiple lines of therapy and recurrence. We analyzed 23 plasma samples to explore how ctDNA levels and mutation profiles correspond to treatment response, remission, and progression. We observed cases where ctDNA paralleled or anticipated clinical course, as well as instances where ctDNA failed to detect radiographically evident disease. These findings illustrate both the potential and the current limitations of ctDNA for disease monitoring in ovarian cancer, underscoring the need for further research to define optimal applications and improve assay performance.

## 2. Materials and Methods

**Study Design and Ethical Approval:** Serial blood samples from patients with ovarian, fallopian tube, or peritoneal carcinoma (collectively termed tubo-ovarian carcinoma) were collected after subjects provided written, informed consent under a prospective study approved by the University of Washington Human Subjects Division Institutional Review Board. Treatment response was assessed by clinical examination, radiographic imaging, and CA-125 level and correlated with serial ctDNA analyses. All patients were followed until death or loss to follow-up.

**Patient Selection and Clinical Background:** Six patients with advanced high-grade serous tubo-ovarian carcinoma (HGSC) were selected for detailed analysis. Selection was based on the availability of longitudinal plasma samples (*N* = 23 total) spanning diagnosis, remission, and recurrence. Clinical histories were reviewed to provide context regarding comorbidities, diagnostic testing, treatment decisions, and outcomes. Diagnostic evaluation typically included pelvic examination, serum CA-125, computed tomography (CT), and tissue confirmation of HGSC by histopathology and immunohistochemistry. Germline and somatic tumor sequencing were performed at diagnosis to identify actionable variants (e.g., BRCA1/2, RAD51D).

Treatment strategies were determined in accordance with clinical guidelines and patient-specific factors. Patients with bulky disease or poor surgical candidacy were offered neoadjuvant chemotherapy, whereas those suitable for upfront cytoreduction underwent primary debulking. Platinum-based doublet chemotherapy formed the backbone of all front-line and platinum-sensitive recurrence regimens, with bevacizumab or poly(ADP-ribose) polymerase (PARP) inhibitors incorporated based on mutational status, treatment response, and trial eligibility. As the disease progressed to platinum resistance, single-agent chemotherapy formed the backbone of treatment with the inclusion of bevacizumab as indicated. The evolution of symptoms—including abdominal bloating, pain, ascites, and treatment-related toxicities—was recorded at each visit. CA-125 and imaging results informed treatment response, while ctDNA was integrated retrospectively for this study.

**Sample Collection and Processing:** Peripheral blood was drawn into Cell-Free DNA Blood Collection Tubes (Streck, La Vista, NE, USA) and processed within 72 h by first fractionating the blood at 1300× *g* for 15 min at room temperature, then the plasma at 16,000× *g* for 10 min at 10 °C. Following centrifugation, plasma supernatant aliquots were stored at −80 °C until further processing.

cfDNA Extraction, Library Preparation, and Sequencing: cfDNA was extracted from 2 to 3 mL of plasma using the QIAamp Circulating Nucleic Acid Kit (Qiagen, Hilden, Germany) according to the manufacturer’s protocol. The extracted cfDNA was quantified using the Qubit dsDNA High Sensitivity Kit (Thermo Fisher Scientific, Waltham, MA, USA). Sequencing libraries were constructed from 5 to 10 ng of cfDNA input using the KAPA HyperPrep kit (Roche Sequencing and Life Science, Wilmington, MA, USA). Each sample was individually barcoded using xGen UDI-UMI adapters (Integrated DNA Technologies, Coralville, IA, USA) and subsequently pooled for multiplexed hybrid capture. Target enrichment was performed using xGen next-generation sequencing (NGS) Hybridization Capture reagents (Integrated DNA Technologies, Coralville, IA, USA), targeting approximately 200 kb of genomic regions across 68 cancer-associated genes. Following hybridization capture, libraries were amplified, purified using AMPure XP beads (Beckman Coulter Life Sciences, Indianapolis, IN, USA), and quantified before sequencing on an Illumina NextSeq 500 (Illumina, San Diego, CA, USA).

**Bioinformatic Analysis:** Tissue-based tumor sequencing was performed as previously described [10]. We performed tumor-informed profiling in which ctDNA samples were interrogated for specific mutations identified in the corresponding tumor tissue. In addition, if ctDNA testing revealed other pathogenic or likely pathogenic variants, these were annotated based on consensus variant classification criteria [11]. This approach ensured that both tumor-confirmed mutations and clinically significant de novo alterations were captured. No clinically significant variants were identified in ctDNA that were not previously identified in tumor tissue. Demultiplexed and aligned reads were processed using fgbio (Fulcrum Genomics, Boulder, CO, USA) to group reads by their unique molecular identifiers (UMIs). Families with fewer than five reads or with more than 5% erroneous bases were discarded. Consensus sequences were generated for each family, and bases without a clear consensus or where more than 10% of reads disagreed were masked to ‘N’. Families with over 10% masked bases were excluded from further analysis. Consensus reads were realigned, and variants were called using VarDict (AstraZeneca-NGS, Cambridge, UK). Alternate alleles were reported if supported by at least one read (base quality score >10) and if the variant allele frequency (VAF) exceeded 0.001%. In addition, large insertions and deletions were identified through manual inspection. The average coverage of uncollapsed UMI reads across all plasma samples was 51,744 (standard deviation 8651), reflecting the total depth of sequencing prior to deduplication. After collapsing reads based on UMIs to account for amplification duplicates, the average coverage was 2234 (standard deviation 930), representing the unique input molecules captured in the assay.

## 3. Results

**Figure 1:**
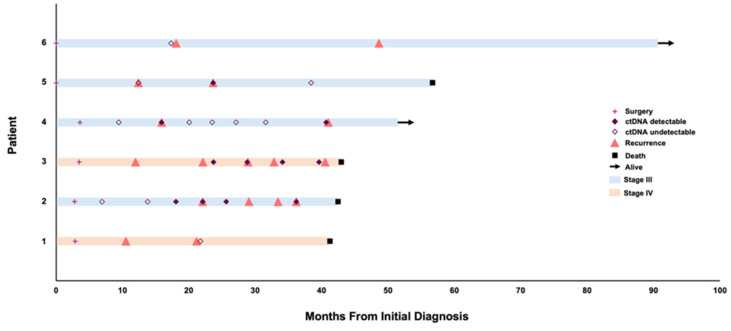
Swimmer plot detailing detection of ctDNA in patients diagnosed with advanced ovarian cancer (*N* = 6).

**Figure 2:**
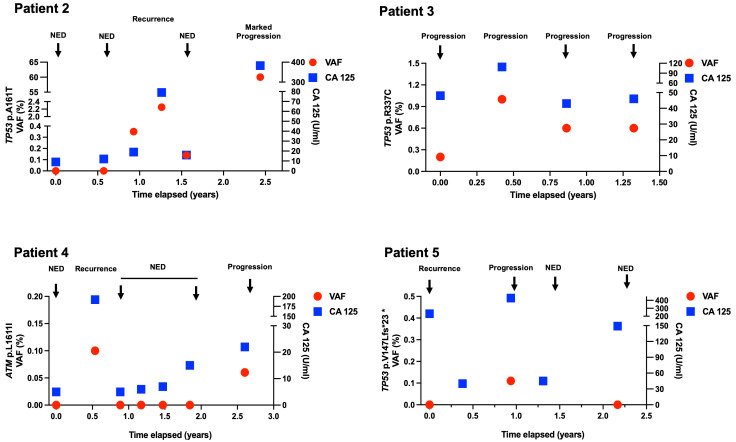
ctDNA and CA-125 dynamics in patients. VAF refers to a specific variant allele frequency monitored for each individual patient. Assay utilized to detect and monitor tissue-informed mutations. (NED-No evidence of disease).

Six patients with HGSC had serial blood samples obtained at diagnosis and through multiple lines of therapy. The swimmor plot in Figure 1 summarizes treatment timelines, ctDNA status, and corresponding clinical outcomes for each patient. Figure 2 provides the ctDNA VAF for patients 2–5 relative to serum CA-125 measurements. Table 1 provides a list of specific variants detected in ctDNA.

### 3.1. Patient 1

A 61-year-old woman with Stage IVA HGSC presented in April 2019 with diffuse peritoneal carcinomatosis, ascites, and malignant pleural effusion causing dyspnea. Given the extent of disease and poor surgical candidacy, she received three cycles of neoadjuvant carboplatin and paclitaxel, followed by interval debulking surgery (IDS). Pathology confirmed high-grade serous carcinoma with widespread peritoneal implants. She then completed three cycles of adjuvant chemotherapy (total of six). Tumor sequencing identified a somatic CDK12 pathogenic variant (PV) with loss of heterozygosity. She had a complete response to therapy, and CA-125 reached a nadir of 7 U/mL.

Following treatment, she achieved complete clinical and radiographic remission, with CA-125 nadir at 7 U/mL. In June 2020, she developed symptomatic recurrence with abdominal distension and radiologic progression. She was treated with cisplatin, doxorubicin, and bevacizumab, achieving a radiologic and biochemical response.

By April 2021, she again progressed with platinum-resistant disease with a rising CA-125 (197 U/mL) and imaging-confirmed disease; however, plasma ctDNA was undetectable at that time. Subsequent regimens included gemcitabine with bevacizumab, weekly paclitaxel with bevacizumab, and later cyclophosphamide with pembrolizumab. Despite multiple salvage therapies, she experienced worsening abdominal pain and cachexia and ultimately succumbed to progressive disease.

### 3.2. Patient 2

A 69-year-old woman with Stage IIIC HGSC presented in October 2017 with extensive carcinomatosis and large-volume ascites. She received three cycles of neoadjuvant carboplatin and paclitaxel, followed by IDS with optimal cytoreduction, then five cycles of adjuvant chemotherapy with concurrent bevacizumab. She achieved a CA-125 nadir of 9 U/mL and complete remission radiographically. Tumor sequencing revealed a complex somatic BRCA2 inversion and a somatic TP53 PV.

Based on homologous recombination deficiency, she was enrolled in a clinical trial of maintenance niraparib and bevacizumab. In April 2019, four months prior to overt progression, ctDNA was detectable at 0.35% VAF despite normal CA-125 (17 U/mL). By August 2019, at first recurrence, ctDNA rose to 2.3% VAF with CA-125 at 79 U/mL and radiographic progression.

She was initially treated with carboplatin with liposomal doxorubicin. After developing platinum-resistant progression, she underwent sequential chemotherapies, including weekly paclitaxel with bevacizumab, gemcitabine, pemetrexed, and topotecan adjusted based on tolerance and progression. Two additional plasma ctDNA collections were mutation-positive during this period. One, in December 2019, at 0.14% VAF while responding to weekly paclitaxel with bevacizumab, with normal CA 125 (19 U/mL) and a CT thereafter confirming response to therapy. The second, in October 2020, with the mutation at 60% VAF, which correlated with marked radiological progression and a CA-125 of 383 (Figure 2), and shortly thereafter, death.

### 3.3. Patient 3

A 74-year-old woman was diagnosed with Stage IVB HGSC in December 2014 following progressive abdominal pain, bloating, and ascites. Initial CT imaging showed widespread carcinomatosis and liver involvement. She received three cycles of neoadjuvant carboplatin and paclitaxel, IDS, and six cycles of carboplatin/gemcitabine/bevacizumab followed by maintenance bevacizumab. CA-125 nadired at <5 U/mL, and she achieved radiographic remission.

Somatic NGS was significant for the TP53 mutation (p.R337C), and no germline mutations were present. By November 2015, she relapsed with platinum-resistant disease and underwent multiple subsequent lines until April 2018, including cyclophosphamide/bevacizumab, liposomal doxorubicin/bevacizumab, a clinical trial of pembrolizumab and carboplatin (NCT03029598), oxaliplatin, topotecan, cisplatin, and nivolumab before succumbing to her disease shortly thereafter. Four plasma ctDNA samples were collected between November 2016 and March 2018, and they all had detectable mutations that correlated with detectable CA-125 and obvious progression on imaging.

### 3.4. Patient 4

A 42-year-old woman presented in June 2017 with abdominal pain and bloating. Imaging showed bulky pelvic sidewall disease and peritoneal spread, leading to a diagnosis of Stage IIIC HGSC. She underwent neoadjuvant carboplatin/paclitaxel, IDS, and six cycles of adjuvant chemotherapy. Tissue sequencing identified a somatic ATM PV.

A ctDNA sample obtained 3.4 months after completion of therapy was negative, consistent with remission (normal CA-125, negative imaging).

In September 2018, she developed a platinum-sensitive recurrence with rising CA-125 (192 U/mL), radiographic progression, and detectable ctDNA.

She underwent treatment with six cycles of carboplatin, gemcitabine, and bevacizumab (followed by maintenance bevacizumab); serial ctDNA samples remained undetectable, correlating again with clinical remission. The final sample in October 2020, however, showed detectable ctDNA, coinciding with a mild CA-125 rise to 22 U/mL but obvious imaging-confirmed progression.

### 3.5. Patient 5

A 69-year-old patient diagnosed with Stage IIIB HGSC in November 2015 underwent primary debulking surgery followed by six cycles of adjuvant carboplatin and paclitaxel, achieving a CA-125 nadir of 25 U/mL. Tumor and germline sequencing identified a germline RAD51D PV and somatic BRCA2 and TP53 PVs. At her first recurrence in November 2016, ctDNA was undetectable despite an elevated CA-125 (231 U/mL) and radiological evidence of disease. Following treatment with carboplatin and liposomal doxorubicin, her CA-125 nadired at 40 U/mL. A plasma sample obtained in November 2017 revealed a ctDNA at TP53 0.11% VAF, concurrent with progression (CA-125 429 U/mL). Later, while off treatment, a plasma sample in January 2019 was negative for ctDNA, even as CA-125 remained modestly elevated (66 U/mL). Notably, CT scans in June and August 2019 did not show radiographic progression despite a marked CA-125 rise (>2000 U/mL), prompting reinitiation of therapy.

### 3.6. Patient 6

A 51-year-old patient with Stage IIIC HGSC in 2015 underwent primary debulking surgery followed by three cycles of carboplatin/paclitaxel and four cycles of combined intravenous/intraperitoneal chemotherapy, achieving a CA-125 nadir of 6 U/mL and complete clinical remission. Two years post-surgery, she developed isolated brain metastases in the posterior fossa, which were treated with 3000 cGy of radiation over 10 fractions. Tumor and blood sequencing revealed somatic PVs in NF1, TP53, and RB1, and a germline PV in RAD51D. Plasma ctDNA remained undetectable during this period, likely due to the blood–brain barrier and consistent with normal serum CA-125 level. Following further treatment, including additional chemotherapy, PARP inhibitor maintenance, and gamma knife for subsequent brain metastases, she ultimately developed leptomeningeal disease and passed away. Of note, she never developed metastases outside the central nervous system.

## 4. Discussion

Our data demonstrate that ctDNA dynamics in advanced-stage ovarian carcinoma frequently mirror clinical status and, in some cases, predict outcomes, particularly when patients have substantial disease burden. Patient 3 displayed persistently detectable ctDNA over multiple lines of therapy, reflecting an aggressive disease course that became refractory to treatment. Similarly, Patients 2, 4, and 5 showed detectable ctDNA levels during periods of obvious progression, which is consistent with prior studies that have demonstrated strong correlations between ctDNA levels, tumor burden, and CA-125 levels in ovarian cancer [12]. Although our cohort was not designed to systematically track ctDNA shifts at each treatment cycle, our experience aligns with the idea that ctDNA can serve as a dynamic biomarker of residual disease burden. Further, in Patient 2, ctDNA levels were elevated (0.35% VAF) months ahead of radiologically evident relapse, at a time when CA-125 remained within normal limits. This lead-time advantage supports the literature suggesting that ctDNA may offer greater sensitivity than CA-125 in detecting molecular relapse [12,13], which, if validated in larger studies, could argue for its use in surveillance in routine clinical care.

Our study employed a high-sensitivity University of Washington ctDNA NGS approach covering 68 cancer-associated genes, using UMIs to minimize background noise [10]. This method offers superior analytical sensitivity compared to early PCR-based assays [13,14] and an expanded mutational breadth beyond ddPCR and limited gene panels. Nonetheless, there were notable cases in which ctDNA failed to correlate with clinical or radiographic progression. Patients 1, 5, and 6 exemplify this phenomenon in vastly different contexts. Patient 1 had undergone two previous rounds of platinum doublet chemotherapy. Patient 5 did not have detectable ctDNA at the time of recurrence, but subsequent samples were positive. Further, Patient 5 had an instance with markedly abnormal CA-125 and symptoms without radiological progression and undetectable ctDNA. Patient 6 had recurrent brain metastases with no evidence of disease anywhere else and a normal CA-125. There could be various reasons for this discordance. Metastatic niches, such as the central nervous system, may produce less ctDNA due to blood–tissue barriers, and very low-volume peritoneal disease may shed insufficient ctDNA. Intratumoral heterogeneity can also influence which mutations are present at recurrence—if the driver mutations that dominate relapsed clones were absent in the original tumor sample, resided in subclones, or were not covered by the targeted panel, they could be missed by ctDNA profiling.

Although it is not universally expressed or reliable, CA-125 remains a valuable adjunct in the management of ovarian cancer. In four of our six patients, ctDNA and CA-125 levels fluctuated in parallel, suggesting that these two biomarkers may complement one another. CA-125 may predict progression before radiological progression, and ctDNA may predict disease progression in the presence of normal CA-125. However, in the recurrent/metastatic setting, they may both be subject to the same clinical limitation. Namely, an asymptomatic rise in CA-125 in the absence of radiological progression is generally not an indication for initiating or changing treatment. It does not change long-term outcomes [15] and introduces treatments and toxicities earlier than necessary, something that we need to be especially mindful of when the intent of therapy is not curative. Presumably, the same limitations apply to ctDNA monitoring.

## 5. Conclusions

ctDNA’s promise in other malignancies should frame the context for its application in ovarian cancer. In several cancer types, ctDNA has been convincingly used to detect minimal residual disease following curative-intent therapy and identify patients at high risk of relapse [16,17,18,19]. Similarly, dynamic changes in ctDNA during neoadjuvant or adjuvant chemotherapy have correlated with response to therapy [20,21]. These findings have led to a remarkable recent success of trials that have used ctDNA to guide and personalize systemic therapy [22,23,24]. It is in this situation that ctDNA may be most useful. As the number of targeted therapies grows, ctDNA could guide risk stratification, choice and duration of therapy, and lead to novel adaptive trials, e.g., in the upfront maintenance setting, ctDNA could guide (1) the optimal duration of PARP inhibitors (NCT06580314—currently evaluating one vs. two years of therapy), (2) precisely identify patients with the highest benefit from bevacizumab, supplementing or replacing the use of KELIM scores [25,26], and (3) as a stratification factor in an adaptive trial of a novel therapy such as cancer vaccines. Large studies to demonstrate its role as a prognostic marker early in the disease course, after neoadjuvant or adjuvant chemotherapy, are key. Ultimately, refining ctDNA-based biomarker strategies and incorporating them early into personalized treatment algorithms could improve long-term outcomes for the thousands of individuals diagnosed with ovarian cancer every year.

## Figures and Tables

**Table 1:** Variants detected in ctDNA.

Case	Variant Detected in ctDNA
**1**	N/A
**2**	*TP53* p.A161T
**3**	*TP53* p.R337C
**4**	*ATM* p.L1611I
**5**	*TP53* p.V147Lfs*23 *
**6**	N/A

N/A = not available, due to undetectable ctDNA

## Data Availability

The raw data supporting the conclusions of this article will be made available by the authors on request.

## Abbreviations

ctDNA	Circulating tumor DNA
OC	Ovarian Cancer
HGSC	High grade Serous Ovarian Cancer

## References

[1] Sant M., Bernat-Peguera A., Felip E., Margelí M. (2022). Role of ctDNA in Breast Cancer. Cancers.

[2] Puccini A., Martelli V., Pastorino A., Sciallero S., Sobrero A. (2023). ctDNA to Guide Treatment of Colorectal Cancer: Ready for Standard of Care?. Curr. Treat. Options Oncol..

[3] Gracie L., Pan Y., Atenafu E.G., Ward D.G., Teng M., Pallan L., Stevens N.M., Khoja L. (2021). Circulating tumour DNA (ctDNA) in metastatic melanoma, a systematic review and meta-analysis. Eur. J. Cancer.

[4] Li S., Noor Z.S., Zeng W., Stackpole M.L., Ni X., Zhou Y., Yuan Z., Wong W.H., Agopian V.G., Dubinett S.M. (2021). Sensitive detection of tumor mutations from blood and its application to immunotherapy prognosis. Nat. Commun..

[5] Verschoor N., Bos M.K., Oomen-de Hoop E., Martens J.W., Sleijfer S., Jager A., Beije N. (2024). A review of trials investigating ctDNA-guided adjuvant treatment of solid tumors: The importance of trial design. Eur. J. Cancer..

[6] Moding E.J., Nabet B.Y., Alizadeh A.A., Diehn M. (2021). Detecting Liquid Remnants of Solid Tumors: Circulating Tumor DNA Minimal Residual Disease. Cancer Discov..

[7] Magbanua M.J.M., Brown Swigart L., Ahmed Z., Sayaman R.W., Renner D., Kalashnikova E., Hirst G.L., Yau C., Wolf D.M., Li W. (2023). Clinical significance and biology of circulating tumor DNA in high-risk early-stage HER2-negative breast cancer receiving neoadjuvant chemotherapy. Cancer Cell.

[8] Provencio M., Serna-Blasco R., Franco F., Calvo V., Royuela A., Auglytė M., Sánchez-Hernández A., Campayo M.d.J., García-Girón C., Dómine M. (2021). Analysis of circulating tumour DNA to identify patients with epidermal growth factor receptor-positive non-small cell lung cancer who might benefit from sequential tyrosine kinase inhibitor treatment. Eur. J. Cancer.

[9] Garlan F., Laurent-Puig P., Sefrioui D., Siauve N., Didelot A., Sarafan-Vasseur N., Michel P., Perkins G., Mulot C., Blons H. (2017). Early Evaluation of Circulating Tumor DNA as Marker of Therapeutic Efficacy in Metastatic Colorectal Cancer Patients (PLACOL Study). Clin. Cancer Res..

[10] Kuo A.J., Paulson V.A., Hempelmann J.A., Beightol M., Todhunter S., Colbert B.G., Salipante S.J., Konnick E.Q., Pritchard C.C., Lockwood C.M. (2020). Validation and implementation of a modular targeted capture assay for the detection of clinically significant molecular oncology alterations. Pract. Lab. Med..

[11] Li M.M., Datto M., Duncavage E.J., Kulkarni S., Lindeman N.I., Roy S., Tsimberidou A.M., Vnencak-Jones C.L., Wolff D.J., Younes A. (2017). Standards and Guidelines for the Interpretation and Reporting of Sequence Variants in Cancer: A Joint Consensus Recommendation of the Association for Molecular Pathology, American Society of Clinical Oncology, and College of American Pathologists. J. Mol. Diagn..

[12] Parkinson C.A., Gale D., Piskorz A.M., Biggs H., Hodgkin C., Addley H., Freeman S., Moyle P., Sala E., Sayal K. (2016). Exploratory Analysis of TP53 Mutations in Circulating Tumour DNA as Biomarkers of Treatment Response for Patients with Relapsed High-Grade Serous Ovarian Carcinoma: A Retrospective Study. PLoS Med..

[13] Kim Y.M., Lee S.W., Lee Y.J., Lee H.Y., Lee J.E., Choi E.K. (2019). Prospective study of the efficacy and utility of TP53 mutations in circulating tumor DNA as a non-invasive biomarker of treatment response monitoring in patients with high-grade serous ovarian carcinoma. J. Gynecol. Oncol..

[14] Alves M.C., Fonseca F.L.A., Yamada A.M.T.D., Barros L.A.D.R., Lopes A., Silva L.C.F.F., Luz A.S., Cruz F.J.S.M., Del Giglio A. (2020). Increased circulating tumor DNA as a noninvasive biomarker of early treatment response in patients with metastatic ovarian carcinoma: A pilot study. Tumor Biol..

[15] Rustin G.J.S., van der Burg M.E.L., Griffin C.L., Guthrie D., Lamont A., Jayson G.C., Kristensen G., Mediola C., Coens C., Qian W. (2010). Early versus delayed treatment of relapsed ovarian cancer (MRC OV05/EORTC 55955): A randomised trial. Lancet.

[16] Tie J., Wang Y., Tomasetti C., Li L., Springer S., Kinde I., Silliman N., Tacey M., Wong H.-L., Christie M. (2016). Circulating tumor DNA analysis detects minimal residual disease and predicts recurrence in patients with stage II colon cancer. Sci. Transl. Med..

[17] Reinert T., Henriksen T.V., Christensen E., Sharma S., Salari R., Sethi H., Knudsen M., Nordentoft I.K., Wu H.-T., Tin A.S. (2019). Analysis of Plasma Cell-Free DNA by Ultradeep Sequencing in Patients With Stages I to III Colorectal Cancer. JAMA Oncol..

[18] Tran H.T., Heeke S., Sujit S., Vokes N., Zhang J., Aminu M., Lam V., Vaporciyan A., Swisher S., Godoy M. (2024). Circulating tumor DNA and radiological tumor volume identify patients at risk for relapse with resected, early-stage non-small-cell lung cancer. Ann. Oncol..

[19] Qiu B., Guo W., Zhang F., Lv F., Ji Y., Peng Y., Chen X., Bao H., Xu Y., Shao Y. (2021). Dynamic recurrence risk and adjuvant chemotherapy benefit prediction by ctDNA in resected NSCLC. Nat. Commun..

[20] Magbanua M.J.M., Swigart L.B., Wu H.T., Hirst G., Yau C., Wolf D., Tin A., Salari R., Shchegrova S., Pawar H. (2021). Circulating tumor DNA in neoadjuvant-treated breast cancer reflects response and survival. Ann. Oncol..

[21] Zhou Q., Gampenrieder S.P., Frantal S., Rinnerthaler G., Singer C.F., Egle D., Pfeiler G., Bartsch R., Wette V., Pichler A. (2022). Persistence of ctDNA in Patients with Breast Cancer During Neoadjuvant Treatment Is a Significant Predictor of Poor Tumor Response. Clin. Cancer Res..

[22] Tie J., Cohen J.D., Lahouel K., Lo S.N., Wang Y., Kosmider S., Wong R., Shapiro J., Lee M., Harris S. (2022). Circulating Tumor DNA Analysis Guiding Adjuvant Therapy in Stage II Colon Cancer. N. Engl. J. Med..

[23] Kotani D., Oki E., Nakamura Y., Yukami H., Mishima S., Bando H., Shirasu H., Yamazaki K., Watanabe J., Kotaka M. (2023). Molecular residual disease and efficacy of adjuvant chemotherapy in patients with colorectal cancer. Nat. Med..

[24] Tie J., Wang Y., Lo S.N., Lahouel K., Cohen J.D., Wong R., Shapiro J.D., Harris S.J., Khattak A., Burge M.E. (2025). Circulating tumor DNA analysis guiding adjuvant therapy in stage II colon cancer: 5-year outcomes of the randomized DYNAMIC trial. Nat. Med..

[25] You B., Robelin P., Tod M., Louvet C., Lotz J.-P., Abadie-Lacourtoisie S., Fabbro M., Desauw C., Bonichon-Lamichhane N., Kurtz J.-E. (2020). CA-125 ELIMination Rate Constant K (KELIM) Is a Marker of Chemosensitivity in Patients with Ovarian Cancer: Results from the Phase II CHIVA Trial. Clin. Cancer Res..

[26] Piedimonte S., Kim R., Bernardini M.Q., Atenafu E.G., Clark M., Lheureux S., May T. (2022). Validation of the KELIM score as a predictor of response to neoadjuvant treatment in patients with advanced high grade serous ovarian cancer. Gynecol. Oncol..

